# Supplementary material for: Increasing Adult Hippocampal Neurogenesis Promotes Resilience in a Mouse Model of Depression
Source: Cells. 2021 Apr 21;10(5):972. doi: 10.3390/cells10050972 (PMC8143348; doi:10.3390/cells10050972)
Supplement: Supplementary file 1 [file cells-10-00972-s001.zip › cells-1178228-supplementary.pdf]

Supplementary materials and methods

Animals

Male iBax mice aged 11 weeks at the start of the experiments were used. The generation and characterization of the *Nes-CreER^{T2}* transgenic mouse line is already described in details (Sahay et al. 2011). The colony of *Nes-CreER^{T2}; Bax*$^{f/f}$ mice was maintained by interbreeding *Nes-CreER^{T2}; Bax*$^{f/f}$ mice and *Bax*$^{f/f}$ mice and were maintained on a mix of C57BL/6 and 129sv genetic background.

To induce CreER^{T2}-mediated recombination of *Bax* in neural stem cells in the adult brain, mice of 11 weeks of age were given 55mg/kg tamoxifen (TAM) intraperitoneally, once a day for 5 consecutive days. Tamoxifen (20 mg ml^{-1}, Sigma, T-5648) stock solution was prepared in corn oil and dissolved each day before the injection. For vehicle-treated mice, 10ml/kg body weight of corn oil was injected intraperitoneally, once a day for 5 consecutive days. Animals were group housed and kept under standard laboratory conditions (12/12h light-dark cycle with lights on at 8:30 p.m. and room temperature 22 ± 2 °C), in enriched cages (PAULA Ferplast 46× 29×25 cm) equipped with one running wheel, tubes and three novel objects added randomly twice every week, during four weeks starting from the first TAM injection. Access to food and water was *ad libitum*. Mice were then divided in two groups depending on whether they had received the unpredictable chronic mild stress regimen (UCMS) or not (non-stressed : NS). Mice subjected to the UCMS regimen were housed in 24×11×12 cm cages without any environmental enrichment from week 5 to 9, while NS mice stayed in enriched cages ; the nineth week, all mice went through behavioral tests and were then sacrificed (figure 2a). All behavioral assessments were conducted during the dark phase. All procedures were compliant with Directive 2010/63/EU guidelines on animal ethics (referral 04808, approved by the ethical committee CEEvdl).

Reward-maze test/ Cookie test

To test for anhedonic traits, mice were subjected to a reward-maze test (Surget et al. 2011; Legrand et al. 2019). A cookie was used as palatable reward in order to test anhedonia. Mice were familiarized with the cookie by giving a sample every two days 1 week before the test to avoid novelty induced hypophagia. Moreover, to minimize environmental neophobia, the test was done under red light and mice were habituated three times to the device during 5 minutes within ten days before the test (inter-test interval 2 days). The apparatus was made of three consecutive chambers (20×20×20 cm) with communicant doors between each. Common food pellets were removed from the cage lid 1 hour before the test. In this test the mouse is placed in the first chamber and the reward is placed at the center of the third one. Once the mouse enters the second chamber, the first door is closed rendering impossible for the mouse to go back. The latency to eat the reward and the consumption of the reward was measured for up to 5 minutes.

Flexibility/inhibition water maze test

This test aims to evaluate 2 aspects of executive functions: cognitive flexibility and inhibitory control. Executive functions represent a set of high-level cognitive processes that support the elaborations and control of complex and adaptive behavioral responses. Among these processes, cognitive flexibility represents the ability to switch between different strategies or behavioral responses, while inhibitory control represents the ability to inhibit or override a

behavioral response previously learnt but that became inefficient or irrelevant, in order to implement more adaptive goal-oriented strategies

The water maze has a plus-shape with 4 arms (N, E, S, W; 38 x 14 X 28 cm) placed in a circular pool (diameter 90cm). The water was maintained at a temperature of 22±2°C. Light intensity at the centre of the device was approximately 100lux. One arm contained a platform (5x5cm) at its extremity placed slightly below the water surface (between 1 and 1.5 cm) so that the platform was not visible directly. Moreover, the platform was placed not too close to the wall of the pool (~5-10cm) in order to avoid that the mouse tries to jump out. The arm N contained a visual cue (a card with strong black/white contrast) and, just above its extremity, a small lamp allowing to illuminate the visual cue at 500 lux. The context in each trial depended on the presence of tactile cue in the water, i.e. presence or absence of small plastic lens (PVC capsules with a diameter of 1-3mm) in the water. The departure took place at the extremity of one of the 3 other arms (E, S, W), head toward the centre of the maze and the position varied from a trial to another.

During the procedure, the mouse had to learn to associate a specific context (presence or absence of the plastic lens in the water) with a specific task (find the platform according to (1) departure position or (2) cue position). Therefore, the animal had to develop 2 different cognitive strategies (egocentric or allocentric) to resolve the problem and find quickly the platform according to the context.
For the association of a context to an egocentric strategy, the mouse had to learn in this context a sequence of directions to find the platform independently of the arm of departure: 'go to the centre of the maze' and then 3 possibilities (1) 'go to the left' (if departure point is W), (2) 'go to the right' (if departure point is E), or (3) 'go straight' (departure from S) in order to find the platform. For a same mouse, the sequence of directions to find the platform (i.e. for the egocentric task) was always the same. This type of task was defined as *'direction'*.
For an association of a context to an allocentric strategy, the mouse had to learn that the platform position was always located at the same place (in the cued arm = arm N) independently of the departure position. This type of task was defined as *'cue'*, because mice had to find the platform according to an external cue present in the arm N, the cue was a card with strong contrast and highly illuminated by a small lamp placed just above the arm.

Accordingly, each mouse during the experiment was subjected to the 2 different contexts, and each of these contexts was associated to a different task. We therefore had 4 possibilities of 'context-task' associations:
- 'w/o lens' + 'direction'
- 'with lens' + 'direction'
- 'w/o lens' + 'cue'
- 'with lens' + 'cue'

The all procedure occurred on several days: 4 days for the learning stage on the first week, then 1 day for the flexibility test and 1 day for the inhibition test on the second week.

In a session of the learning stage, the mice underwent 4 blocks of 5 trials/block. When all the mice have performed block 1, we started block 2, and so forth until block 4 was finished. The time between 2 blocks corresponded to the time to test all the mice in this block. For the

flexibility and inhibition test, the procedure was similar excepted that mice performed 6 blocks.
From a trial to another, the departure position varied in one of the three departure arms (E, S and W) in a semi-random manner and in a way that the number of departures into each arm was well-balanced.
We let the mouse to recover on the platform between each trial for a duration of 30 seconds minimum and 60 seconds maximum (inter-trial interval = ITI). The maximum time for each trial is 1 minute; if the mouse had not reached the platform within 1 minute, the experimenter musted gently lead the mouse to the platform.

Days 1-2 The mice learned one of the two strategies in the presence of the first tactile cue (context A – task 1).

Days 3-4 The mice learned the second strategy in the presence of the other tactile cue (context B – task 2).

As a consequence, the mice associated a specific context to a specific task and had to develop an egocentric vs. allocentric strategy depending on the context.
One week following the learning phase, mice were tested for flexibility and inhibition capacities.

Day 12, The mice underwent the ***flexibility test***. The mice had to alternate the task learnt at days 1-2 and the task learnt at days 3-4. Accordingly, the presence of the tactile cue and the task to perform varied from a block to another. There was an alternation between 'Context A – Task 1' and 'Context B – Task 2': blocks 1-3-5 for the context A and blocks 2-4-6 for context B. For each trial, the latency to reach the platform and the number of perseverative /interfering errors (exploration of the arm that should have contained the platform in the other context) were recorded.

Day 13, The mice underwent the ***inhibition test***. In this stage the Context B was now associated with Task 1 (previously associated to Context A). Accordingly, the mice had to inhibit the behavioral response previously learnt in context B which had become irrelevant, therefore, this task measures reversal learning capacities. For each trial, the latency to reach the platform and the number of perseverative/interfering errors (exploration of the arm that should have contained the platform as it was when context B was associated to Task 2) were recorded

Immunohistochemistry

At the end of the experiments, mice were injected with an overdose of pentobarbital solution (Dolethal®, 100mg/kg), then transcardially perfused with 50 ml of heparine saline solution to remove blood, followed by 100ml of 4% paraformaldehyde (PFA) in phosphate buffer 0,1M solution to fix the brain. After that, brains were extracted and placed overnight in PFA 4% solution, then cryoprotected in sucrose solution (20%) and stored at 4 C°. For immunochemistry, brains were cut into 30-µm coronal sections with a cooled microtome (−20° Celsius, Leica CM 3050 S).
In order to quantify the AHN, a free-floating immunochemistry against doublecortine (DCX), a marker of immature neurons, has been performed. First, a heat antigen retrieval in citrate

buffer (10mM, pH=6) was performed on brain slices for 10 min at 95 C°. After, brain sections were rinsed 10 minutes with phosphate-buffered saline 0,1M (PBS) then, sections were incubated in blocking solution, 0,5 % triton X-100 and 4% normal donkey serum (NDS) in PBS 0,1M for 90 minutes at room temperature. Incubation with primary antibodies was carried out at 4 C° for 48 hours (DCX antibody 1/750 dilution, ab18723; Abcam). Following 3 washes of 10 minutes each in PBS, sections were incubated with secondary antibodies (Donkey anti-mouse Alexa Fluor555, 1/500 dilution, ab150106; Abcam) in blocking solution for 2 hours at room temperature. Finally, slices were mounted onto slides, recover of Vectashield® mounting medium and stored at 4 C° .

The immunolabelled sections were observed under a Zeiss Z.2 Imager microscope in emitted-light mode, and DCX labelled cells were counted in the DG, at X20 magnificence.
An unbiased and blinded protocol was used to count the DCX labelled cells in the granule cell layer of the dentate gyrus along the septotemporal axis. For quantification, 7 matched sections have been selected for each mouse (4 sections for dorsal hippocampus from bregma -1,3 to -1.8 mm, and 3 sections for ventral hippocampus from bregma -3.3 to -3,6 mm) and DCX cells were expressed as normalized cellular densities (DCX cells/mm^2). Additionally, to evaluate the maturation, DCX cells with at least tertiary dendrites were counted, the maturation index was then expressed as the ratio of DCX cells with at least tertiary dendrites over the total number of DCX cells.

Statistical supplementary information

Test	Measurement	Statistical test	Comparisons	DDL	F	p-value
TST	Immobility	Two-way ANOVA	Stress	1 , 30	7,039	**0,013**
			Treatment	1 , 30	3,302	0,079
			Stress*Treatment	1 , 30	1,115	0,299
		Post-hoc	NS-veh v UCMS veh			**0,015**
			NS-tam v UCMS tam			0,260
			NS-veh v NS-tam			0,613
			UCMS-veh v UCMS tam			**0,040**
NSF	Latency to smell	Two-way ANOVA	Stress	1 , 45	0,051	0,823
			Treatment	1 , 45	1,716	0,197
			Stress*Treatment	1 , 45	0,704	0,406
	Consumption	Two-way ANOVA	Stress	1 , 45	0,293	0,591
			Treatment	1 , 45	1,429	0,238
			Stress*Treatment	1 , 45	0,128	0,722
Nest	Score at 5h	Two-way ANOVA	Stress	1 , 45	7,103	**0,011**
			Treatment	1 , 45	1,585	0,215
			Stress*Treatment	1 , 45	8,299	**0,006**
		Post-hoc	NS-veh v UCMS veh			**0,000**
			NS-tam v UCMS tam			0,269
			NS-veh v NS-tam			**0,005**
			UCMS-veh v UCMS tam			0,248
Light/Dark box	Dark frequency	Two-way ANOVA	Stress	1 , 45	14,269	**0,000**
			Treatment	1 , 45	0,505	0,481
			Stress*Treatment	1 , 45	0,576	0,452
		Post-hoc	NS-veh v UCMS veh			**0,003**
			NS-tam v UCMS tam			**0,037**
			NS-veh v NS-tam			0,300
			UCMS-veh v UCMS tam			0,973
	Dark duration	Two-way ANOVA	Stress	1 , 45	0,970	0,330
			Treatment	1 , 45	0,605	0,441
			Stress*Treatment	1 , 45	0,024	0,878
Splash	Grooming duration	Two-way ANOVA	Stress	1 , 45	1,604	0,212
			Treatment	1 , 45	0,792	0,378
			Stress*Treatment	1 , 45	0,057	0,813
Cookie test	Consumption	Two-way ANOVA	Stress	1 , 41	0,856	0,360
			Treatment	1 , 41	0,043	0,837
			Stress*Treatment	1 , 41	0,032	0,860
	Latency to eat	Two-way ANOVA	Stress	1 , 41	1,867	0,179
			Treatment	1 , 41	5,593	**0,023**
			Stress*Treatment	1 , 41	2,506	0,121
		Post-hoc	NS-veh v UCMS veh			**0,037**
			NS-tam v UCMS tam			0,883
			NS-veh v NS-tam			0,586
			UCMS-veh v UCMS tam			**0,008**
Water Maze/ Flexibility	Average latency	Two-way ANOVA	Stress	1 , 27	0,060	0,809
			Treatment	1 , 27	3,267	0,082
			Stress*Treatment	1 , 27	1,593	0,218
	Total perseverative failures	Two-way ANOVA	Stress	1 , 27	0,008	0,931
			Treatment	1 , 27	5,183	**0,031**
			Stress*Treatment	1 , 27	0,084	0,774
		Post-hoc	NS-veh v UCMS veh			0,807
			NS-tam v UCMS tam			0,877
			NS-veh v NS-tam			0,162
			UCMS-veh v UCMS tam			0,087

**Water maze/Inhibition**	Average latency	Two-way ANOVA	Stress	1 , 27	11,910	**0,002**
			Treatment	1 , 27	12,405	**0,002**
			Stress*Treatment	1 , 27	9,152	**0,005**
		Post-hoc	NS-veh v UCMS veh			**< 0,001**
			NS-tam v UCMS tam			0,744
			NS-veh v NS-tam			0,722
			UCMS-veh v UCMS tam			**< 0,001**
	Total perseverative failures	Two-way ANOVA	Stress	1 , 27	5,514	**0,026**
			Treatment	1 , 27	6,432	**0,017**
			Stress*Treatment	1 , 27	3,030	0,093
		Post-hoc	NS-veh v UCMS veh			**0,012**
			NS-tam v UCMS tam			0,642
			NS-veh v NS-tam			0,570
			UCMS-veh v UCMS tam			**0,006**
**DCX labelled cells**	Total hippocampus	Two-way ANOVA	Stress	1 , 12	0,089	0,771
			Treatment	1 , 12	4,755	**0,050**
			Stress*Treatment	1 , 12	0,174	0,684
		Post-hoc	NS-veh v UCMS veh			0,623
			NS-tam v UCMS tam			0,934
			NS-veh v NS-tam			0,236
			UCMS-veh v UCMS tam			0,091
	Dorsal hippocampus	Two-way ANOVA	Stress	1 , 12	4,968	**0,046**
			Treatment	1 , 12	3,922	0,071
			Stress*Treatment	1 , 12	0,138	0,717
		Post-hoc	NS-veh v UCMS veh			0,091
			NS-tam v UCMS tam			0,213
			NS-veh v NS-tam			0,277
			UCMS-veh v UCMS tam			0,122
	Ventral hippocmpus	Two-way ANOVA	Stress	1 , 12	2,942	0,112
			Treatment	1 , 12	3,948	0,070
			Stress*Treatment	1 , 12	0,150	0,705
**Maturation index**	Total hippocampus	Two-way ANOVA	Stress	1 , 12	1,180	0,299
			Treatment	1 , 12	19,322	**0,001**
			Stress*Treatment	1 , 12	0,000	0,991
		Post-hoc	NS-veh v UCMS veh			0,462
			NS-tam v UCMS tam			0,453
			NS-veh v NS-tam			0,009
			UCMS-veh v UCMS tam			**0,009**
	Dorsal hippocampus	Two-way ANOVA	Stress	1 , 12	2,260	0,159
			Treatment	1 , 12	7,186	**0,020**
			Stress*Treatment	1 , 12	0,013	0,912
		Post-hoc	NS-veh v UCMS veh			0,345
			NS-tam v UCMS tam			0,276
			NS-veh v NS-tam			0,072
			UCMS-veh v UCMS tam			0,094
	Ventral hippocampus	Two-way ANOVA	Stress	1 , 12	0,005	0,946
			Treatment	1 , 12	40,715	**<0,0001**
			Stress*Treatment	1 , 12	0,029	0,867
		Post-hoc	NS-veh v UCMS veh			0,868
			NS-tam v UCMS tam			0,944
			NS-veh v NS-tam			**0,001**
			UCMS-veh v UCMS tam			**0,001**

**Water Maze/ Inhibition**	Latency to find plaform	Two-way ANOVA with repeated measures	Stress	1 , 27	8,970	**0,005**
			Treatment	1 , 27	11,493	**0,002**
			Block	5 , 135	51,808	**< 0,0001**
			Stress*Treatment	1 , 27	9,152	**0,005**
			Stress*Block	5 , 135	5,619	**< 0,0001**
			Treatment*Block	5 , 135	2,558	**0,029**
			Stress*Treatment*Block	5 , 135	3,586	**0,004**
		Post-hoc / Block 1	NS-veh v UCMS veh			**< 0,001**
			NS-tam v UCMS tam			0,621
			NS-veh v NS-tam			0,988
			UCMS-veh v UCMS tam			**< 0,001**
		Post-hoc / Block 2	NS-veh v UCMS veh			**0,006**
			NS-tam v UCMS tam			0,697
			NS-veh v NS-tam			0,944
			UCMS-veh v UCMS tam			**0,014**
		Post-hoc / Block 3	NS-veh v UCMS veh			0,221
			NS-tam v UCMS tam			0,757
			NS-veh v NS-tam			0,705
			UCMS-veh v UCMS tam			0,196
		Post-hoc / Block 4	NS-veh v UCMS veh			0,197
			NS-tam v UCMS tam			0,975
			NS-veh v NS-tam			0,847
			UCMS-veh v UCMS tam			0,130
		Post-hoc / Block 5	NS-veh v UCMS veh			0,538
			NS-tam v UCMS tam			0,692
			NS-veh v NS-tam			0,896
			UCMS-veh v UCMS tam			0,379
		Post-hoc / Block 6	NS-veh v UCMS veh			0,551
			NS-tam v UCMS tam			0,975
			NS-veh v NS-tam			0,717
			UCMS-veh v UCMS tam			0,323
**Water Maze/ Flexibility**	Latency to find plaform	Two-way ANOVA with repeated measures	Stress	1 , 27	0,001	0,970
			Treatment	1 , 27	3,072	0,088
			Block	5 , 135	16,753	**< 0,0001**
			Stress*Treatment	1 , 27	1,593	0,215
			Stress*Block	5 , 135	0,310	0,907
			Treatment*Block	5 , 135	1,884	0,099
			Stress*Treatment*Block	5 , 135	0,308	0,907
**Coat states**	Score	Two-way ANOVA with repeated measures	Stress	1 , 45	421,272	**< 0,0001**
			Treatment	1 , 45	0,002	0,968
			Week	5 , 225	66,674	**< 0,0001**
			Stress*Treatment	1 , 45	0,028	0,867
			stress*Week	5 , 225	68,254	**< 0,0001**
			Treatment*Week	5 , 225	0,421	0,834
			Stress*Treatment*Week	5 , 225	0,320	0,901
		Post-hoc / Week 1	NS-veh v UCMS veh			1,000
			NS-tam v UCMS tam			1,000
			NS-veh v NS-tam			1,000
			UCMS-veh v UCMS tam			1,000
		Post-hoc / Week 2	NS-veh v UCMS veh			**< 0,0001**
			NS-tam v UCMS tam			**0,000**
			NS-veh v NS-tam			1,000
			UCMS-veh v UCMS tam			0,695
		Post-hoc / Week 3	NS-veh v UCMS veh			**< 0,0001**
			NS-tam v UCMS tam			**< 0,0001**
			NS-veh v NS-tam			1,000
			UCMS-veh v UCMS tam			0,976
		Post-hoc / Week 4	NS-veh v UCMS veh			**< 0,0001**
			NS-tam v UCMS tam			**< 0,0001**
			NS-veh v NS-tam			1,000
			UCMS-veh v UCMS tam			0,844
		Post-hoc / Week 5	NS-veh v UCMS veh			**< 0,0001**
			NS-tam v UCMS tam			**< 0,0001**
			NS-veh v NS-tam			0,844
			UCMS-veh v UCMS tam			0,763
		Post-hoc / Week 6	NS-veh v UCMS veh			**<0,0001**
			NS-tam v UCMS tam			**< 0,0001**
			NS-veh v NS-tam			1,000
			UCMS-veh v UCMS tam			0,424
